# Improving marine disease surveillance through sea temperature monitoring, outlooks and projections

**DOI:** 10.1098/rstb.2015.0208

**Published:** 2016-03-05

**Authors:** Jeffrey Maynard, Ruben van Hooidonk, C. Drew Harvell, C. Mark Eakin, Gang Liu, Bette L. Willis, Gareth J. Williams, Maya L. Groner, Andrew Dobson, Scott F. Heron, Robert Glenn, Kathleen Reardon, Jeffrey D. Shields

**Affiliations:** 1 Department of Ecology and Evolutionary Biology, Cornell University, Ithaca, NY 14853, USA; 2 Laboratoire d'Excellence “CORAIL” USR 3278 CNRS—EPHE, CRIOBE, Papetoai, Moorea, Polynésie Française; 3 Atlantic Oceanographic and Meteorological Laboratory, NOAA, 4301 Rickenbacker Causeway, Miami, FL 33149, USA; 4 Cooperative Institute for Marine and Atmospheric Studies, Rosenstiel School of Marine and Atmospheric Science, University of Miami, 4600 Rickenbacker Causeway, Miami, FL 33149, USA; 5 NESDIS Center for Satellite Applications and Research, NOAA Coral Reef Watch, 5830 University Research Center, E/RA3, College Park, MD 20740, USA; 6 Australian Research Council (ARC) Centre of Excellence for Coral Reef Studies, James Cook University, Townsville, Queensland 4811, Australia; 7 College of Marine and Environmental Sciences, James Cook University, Townsville, Queensland 4811, Australia; 8 School of Ocean Sciences, Bangor University, Menai Bridge, Anglesey LL59 5AB, UK; 9 Centre for Veterinary Epidemiological Research, Atlantic Veterinary College, University of Prince Edward Island, Charlottetown, Prince Edward Island, Canada C1A 4P3; 10 Ecology and Evolutionary Biology, Princeton University, Princeton, NJ 08540, USA; 11 Marine Geophysical Laboratory, Physics Department, College of Science, Technology and Engineering, James Cook University, Townsville, Queensland 4814, Australia; 12 Energy and Environmental Affairs, Division of Marine Fisheries, Commonwealth of Massachusetts, 30 Emerson Avenue, Gloucester, MA 01931, USA; 13 Department of Marine Resources, Maine, 21 State House Station, Augusta, ME 04333, USA; 14 College of William and Mary, Virginia Institute of Marine Science, Gloucester Point, VA 23062, USA

**Keywords:** climate change, *Homarus americanus*, epizootic shell disease, marine disease, predictive tools, resource management

## Abstract

To forecast marine disease outbreaks as oceans warm requires new environmental surveillance tools. We describe an iterative process for developing these tools that combines research, development and deployment for suitable systems. The first step is to identify candidate host–pathogen systems. The 24 candidate systems we identified include sponges, corals, oysters, crustaceans, sea stars, fishes and sea grasses (among others). To illustrate the other steps, we present a case study of epizootic shell disease (ESD) in the American lobster. Increasing prevalence of ESD is a contributing factor to lobster fishery collapse in southern New England (SNE), raising concerns that disease prevalence will increase in the northern Gulf of Maine under climate change. The lowest maximum bottom temperature associated with ESD prevalence in SNE is 12°C. Our seasonal outlook for 2015 and long-term projections show bottom temperatures greater than or equal to 12°C may occur in this and coming years in the coastal bays of Maine. The tools presented will allow managers to target efforts to monitor the effects of ESD on fishery sustainability and will be iteratively refined. The approach and case example highlight that temperature-based surveillance tools can inform research, monitoring and management of emerging and continuing marine disease threats.

## 1. Introduction

Media coverage of human emergencies caused by heat waves, severe tropical storms, blizzards, tornados, bush fires, earthquakes and tsunamis overshadow the fact that acute events and chronic, gradual changes in the environment also cause wildlife emergencies. Such emergencies include disease outbreaks. Models and systems that monitor, forecast and project environmental conditions have thus far rarely been used to develop surveillance tools for wildlife diseases [1]. However, significant investments in forecasting models have created the capability to develop such surveillance tools and climate change now provides greater impetus for their use [2]. This is especially the case in the marine environment where the incidence of disease has been on the rise [3–5] and some diseases, like those of corals, are expected to increase with warming [1]. Further, many marine host–pathogen systems are temperature sensitive [3,5,6], and sea temperature can easily be monitored and modelled [7–10].

In the ocean, temperature-based disease surveillance tools have been developed for tropical corals to monitor and forecast coral bleaching [7,11–13]. Thermal coral bleaching is a physiological, non-infectious disease, caused by breakdown and ejection of the algal symbionts when high temperatures persist [14]. The US-based NOAA Coral Reef Watch (CRW) programme maintains a website that hosts near real-time monitoring tools, seasonal outlooks and long-term projections of bleaching conditions under climate change ([7]; coralreefwatch.noaa.gov). These tools inform resource managers of bleaching events and monitor the scale and severity of the events [7,15]. In addition, links between measures of thermal stress and the group of infectious diseases called ‘white syndromes’ (WS) in corals have been used to develop near real-time monitoring tools and seasonal outlooks of disease-conducive conditions [1,16,17]. These tools have been adopted to form an early warning system for the Australian Great Barrier Reef Marine Park Authority's Coral Disease Response Plan, one of the few formal marine disease response plans for diseases linked directly to temperature [18]. Long-term projections have also been developed for identifying climate conditions that increase coral disease susceptibility and pathogen abundance and virulence [1].

The bleaching and WS examples from ecosystems like coral reefs that are already impacted by rising temperatures demonstrate that temperature-based disease surveillance tools can inform research, monitoring and management. Such tools have four primary applications: (i) target efforts to use rapidly advancing diagnostic tools to confirm disease presence or assess prevalence; (ii) target research and monitoring that builds understanding of the role of environmental conditions in disease aetiology; (iii) target management actions that mitigate disease or downstream impacts (e.g. closing areas to human activity); and (iv) raise awareness of disease risk either to educate stakeholders or build political or social will and acceptance of planned research, monitoring and management actions [18]. There are other environmental factors that can influence the likelihood of marine disease outbreaks (e.g. pollution, eutrophication or salinity), independently or synergistically with temperature [19,20]. However, temperature-based products are a logical starting point and launching pad for development of surveillance tools for marine diseases based on monitoring and projecting environmental conditions. Among the host–pathogen–environment relationships, there are many cases in which the role of temperature has been most clearly elucidated. There are also established data archives for sea temperature and weather and climate models that can be used to develop seasonal outlooks and projections.

Our objective is to describe how the scientific and management communities can develop temperature-based surveillance tools for marine diseases. We first describe the process by which these tools are developed, explaining the interplay between research into temperature–disease relationships and the process of developing and refining surveillance tools. We then present a case study showing our process and initial development of surveillance tools for epizootic shell disease (ESD) in the American lobster, *Homarus americanus*.

## 2. Developing temperature-based disease surveillance tools

**Figure 1.**
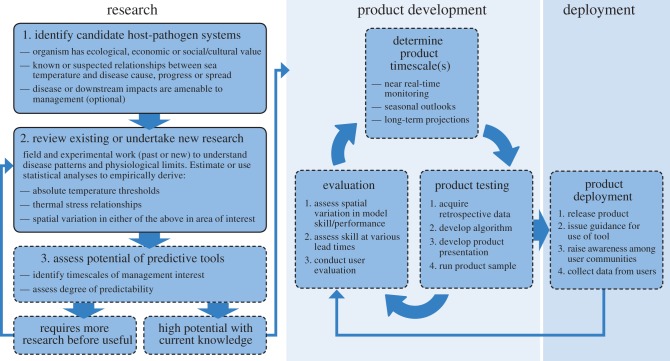
Process for development of temperature-based disease surveillance tools. The three-part research process concludes with assessing disease predictability and then either proceeding with product development and deployment or continuing to undertake research. Product deployment is not an endpoint as tools are iteratively evaluated and continually improved through research and end-user consultation.

Disease surveillance tools require research and ‘product’ development (figure 1). The research component consists of understanding disease–temperature relationships and underpins product development. After the tools are deployed (figure 1), research and product development are ongoing and result in improved versions of the surveillance tools with greater predictive ability.

### (a) Research

The research component for surveillance tools includes identifying candidate diseases and then describing disease links with temperature and assessing disease predictability (figure 1).

#### (i) Identifying candidate diseases

We identified 24 host–pathogen systems that could be candidates for the development of temperature-based surveillance tools using the paper authors as an expert focus group (table 1 and electronic supplementary material, table S1). Here, candidate host–pathogen systems had to meet the following criteria: (i) the host–pathogen system is well known, and temperature is a primary factor associated with outbreaks; (ii) outbreaks of the pathogen have severe impacts (at any spatial scale) on host populations, with ecological, economic or social/cultural consequences of concern to resource managers or the scientific community; and (iii) temperature-based surveillance tools for the disease could help resource managers mitigate disease or downstream impacts or could otherwise benefit scientists, stakeholders or community members. The list of 24 candidate host–pathogen systems was then split into nine that are good candidates (table 1) and 15 that are potential candidates. Experimental and field-based evidence for host–pathogen systems considered good candidates suggests that initial versions of surveillance tools could be produced with limited further research. Potential candidates could be good candidates depending on the outcomes of further research, including understanding synergism of other stressors with temperature (electronic supplementary material, table S1). This expert-based classification was based on the strength of evidence linking temperature to disease causation, progression or spread.

**Table 1.** Host–pathogen systems identified as good candidates (viable now) for developing temperature-based surveillance tools, based on expert opinion. For these systems, disease and downstream impacts are amenable to management actions and the role of temperature in the disease aetiology is well established. This list shows the breadth of host–pathogen systems that are good candidates for surveillance tools but is not expected to be exhaustive. See electronic supplementary material, table S1 for an overview of potential candidates; these could be good candidates for developing temperature-based surveillance tools depending on the outcomes of future research.

hosts	species	causative agent or disease name	region	references
sponges				
sponges	comm. dictyoceratids, *Ircinia* spp*., Sarcotragus* spp*.*	microbial consortium	Mediterranean sea	[21]
corals				
corals	several species	white syndromes and black band disease	global	[1,16,17,22–24]
corals	*Paramuricea clavata*	microbial consortium	Mediterranean sea	[25]
molluscs				
oyster	*Crassostrea virginica*	*Perkinsus marinus*	Mid-Atlantic USA	[26–29]
	*Crassostrea virginica*	MSX - *Haplosporidium nelsoni*	Mid-Atlantic USA	[30,31]
	*Crassostrea virginica*	*Vibrio* spp*.*	human pathogen	[32,33]
Pacific oyster	*Crassostrea gigas*	*Vibrio splendidus*	Western Europe	[34,35]
abalone	*Haliotis rubra*	*Perkinsus olseni*	Australia	[36]
crustaceans				
lobster	*Homarus americanus*	epizootic shell disease	NE N America	[37,38]
vertebrates				
salmon	salmonids	salmon louse	Canada, US, N Europe, Chile (farmed salmon only)	[39–41]
plants				
eelgrass	*Zostera* spp*.*	*Labyrinthula* spp*., L. zosterae*	N America, Europe	[42–44]

The nine host–pathogen systems considered good candidates include: *Vibrio* spp*.* [32,33], MSX [30,31] and *Perkinsus marinus* [26–29] in the oyster *Crassostrea virginica*; a microbial consortium causing black band disease (BBD) in stony tropical corals [23,24]; *Labyrinthula zosterae* causing eelgrass wasting disease in the temperate eelgrass *Zostera marina* [42–44]; and the suite of bacteria causing ESD in the American lobster, *Homarus americanus* [37,38,45,46] (see table 1 for a full list). The 15 host–pathogen systems classified as potential candidates include: *Hematodinium perezi* in the blue crab *Callinectes sapidus* [47–49]; IHNV in Canadian salmon [50]; and wasting disease in several species of sea stars [51] (electronic supplementary material, table S1). The lists of good and potential candidates show the breadth of host–pathogen systems for which temperature-based disease surveillance tools could be developed.

#### (ii) Describing disease links with temperature and assessing disease predictability

**Figure 2.**
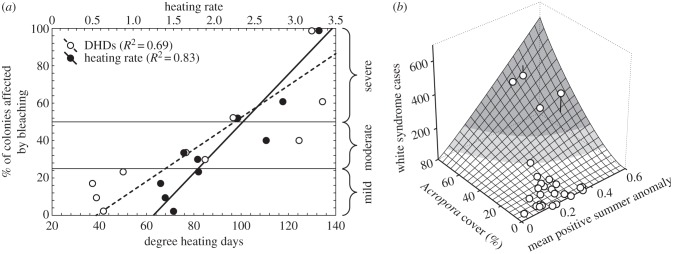
Examples from coral reefs relating bleaching observations and the diseases known as ‘white syndromes' to thermal stress metrics. The metrics here are degree heating days (DHDs) and the mean positive summer anomaly (‘heating rate’ on left), both of which represent stress accumulation above a baseline (average of maximum warm season temperatures). Elucidating these host–disease temperature relationships is the foundation upon which temperature-based disease surveillance tools are built ((*a*) is an adapted version of fig. 3 in [25] and (*b*) is reproduced here with permission from *Coral Reefs* [17]).

Temperature can alter host–pathogen interactions in many ways, and understanding the relationship between temperature and disease risk (emergence or disease severity) is the foundation upon which temperature-based surveillance tools are built. Researchers can gain this knowledge through laboratory experiments with different temperature treatments that start with both diseased and asymptomatic individuals. Field surveys can also be used to relate *in situ* observations of spatio-temporal disease patterns with *in situ*, remotely sensed or modelled temperature data. For laboratory experiments and field surveys, the goal is to gather data that can be used in statistical models to understand the shape of the relationship between temperature and disease risk (examples, figure 2). This will be driven by the shape of the temperature–performance curve for the host–pathogen system. Biological rates typically show unimodal responses to temperature, a generality supported by two recent meta-analyses [52,53]. Metabolic reaction rates tend to increase exponentially up to an optimal temperature, beyond which responses often decline more rapidly than they rise, meaning these relationships are often left skewed (review in [54]). As an example, the unimodal relationship between temperature and malaria transmission is shown within Mordecai *et al.* [54].

Quantifying temperature–performance curves for marine diseases is notoriously difficult in the field. Available temperature measurements may not be representative of the ‘microniches' occupied by species of interest. Species may be regulating their exposure to temperatures (e.g. by inducing ‘behavioural fevers’ or staying in shady spots), or the timeframe over which temperature conditions need to be characterized may be unknown. Further challenging this endeavour are lag effects, cumulative effects or effects of temperature ‘hot or cold snaps’ not captured by the temporal resolution of available temperature data. Finally, temperature often covaries with other factors that change seasonally, such as salinity or community composition; these may need to be accounted for in a model before a temperature effect can be detected. For all of these reasons, statistical model fitting, informed by knowledge of the biology of the system, is often the best approach to determining whether and which temperature conditions can be used to forecast disease risk.

In undertaking model fitting for linear or nonlinear models, a range of variables that describe temperature conditions can be examined based on: (i) absolute temperatures; (ii) temperature anomalies relative to a regional baseline; (iii) the accumulation of temperature stress above a threshold representing the regional climatological average or average maximum temperatures (usually over a 10+ year timeframe); or (iv) temperature variability. Each of these four types of predictor variables can be quantified over a range of timescales and can include consideration of lag effects (i.e. by shifting the time period for which the temperature condition is quantified relative to disease emergence or manifestation of the disease severity level of concern). In all, dozens of candidate predictor variables may be considered when fitting models. There is no general hierarchy in the ability of the four types of temperature variables to predict disease; the merit of each will vary among host–pathogen systems. Example response variables include prevalence, transmission rate, progression rate and severity. The selected response variable (described from here as simply ‘disease risk’) is then predicted using the predictor variable(s) that have links to plausible mechanisms, and that contribute to the top performing models; i.e. they best explain variance in the onset, progression or disease transmission documented in the laboratory or from field observations.

The relationships between temperature and non-infectious coral bleaching are assumed to be linear. The widely applied thermal stress metric for coral bleaching, the ‘degree heating week (DHW) or day’, mathematically represents the accumulation of temperatures above typical summer maxima [55]. The DHW metric outperforms excedance of, for example, any absolute temperature when predicting spatial patterns in bleaching response severity (e.g. per cent of corals affected or average per cent of colony bleached) [56,57]. DHW predicts the bleaching response, because high temperatures have to persist for bleaching (i.e. the loss of algal symbionts) to be severe enough for paling of the corals to be visible. The DHW metric can predict the stress response despite not accounting for cool days and capturing what these reprieves from temperature stress may mean for bleaching risk. Knowing a/the temperature–disease mechanism gives researchers more confidence as to which predictor variables to examine or prioritize in model frameworks and more confidence in the results. However, in many cases, temperature–disease mechanisms will be unknown or poorly understood. In these cases, the model-fitting exercises undertaken will be largely exploratory; correlations found can still be used to develop surveillance tools though confidence may be lower in these until research advances understanding of temperature–disease mechanisms.

The final research step in developing surveillance tools is model validation. To validate a model, one tests how well hindcasts of disease risk based on statistical models predict instances in which the disease occurred or did not occur. The key here is to consider the degree of predictability in the context of the anticipated purpose(s) of the tool. For example, higher degrees of predictability might be required to warrant making management decisions that limit or shape human activities (e.g. close a fishery) than to inform citizen science programmes aimed at increasing understanding of disease via outreach. After assessing predictive ability, a decision is made as to whether to continue to undertake research into the temperature–disease relationship or to proceed with developing surveillance tools (see step 3, figure 1). In many cases, developing first draft (or ‘beta’) versions of surveillance tools will be warranted solely to target research and monitoring efforts that can increase understanding of disease aetiology.

### (b) Product development and deployment

The development of temperature-based surveillance tools is a cycle of determining the product timescale(s), testing, deploying and then iteratively evaluating the temperature–disease relationship, uncertainty and forecast presentation and utility (see process description in figure 1). Temperature-based surveillance tools can assess disease-conducive conditions on various timescales: in near real-time, as seasonal outlooks with one to 12 month (typically less than or equal to six months) lead times, and as long-term projections that typically range from 1 to 85 years (i.e. pre-2100). Each product type or timescale has a different benefit or purpose with respect to how research, monitoring and management decisions can be informed.

(1) Near real-time monitoring involves compilation of very recent remotely sensed, *in situ* or modelled sea surface temperature data (from recent days or weeks). Near real-time monitoring tools inform rapid responses by helping make reactive decisions on scales of days to weeks to target research, monitoring or management [18,58]. For example, near real-time monitoring of temperatures that promote the group of coral diseases ‘WS’ can be used to target research and monitoring that can increase understanding of WS transmission and the role of coral colony density.
(2) Seasonal outlooks use seasonal climate models that take account of recent conditions and produce forecasts of how sea temperatures may change in the coming months. Outlooks can inform reactive decisions as well as near-term planning. A new management action may be implemented or an existing action changed to prepare for a disease outbreak event predicted to occur. For example, coral reef areas can be closed to limit human activities (e.g. diving and fishing) that would further stress organisms affected by diseases such as BBD or that could cause disease to spread among areas [59,60]. Outlooks can be presented within communications products that explain disease risk conditions to senior decision-makers or the public. Such communication based on outlook results can mobilize resources or generate the political or social will required to respond to events when they occur.
(3) Long-term projections are developed using climate models driven by emissions scenarios established by the Intergovernmental Panel on Climate Change. Output units are typically in years, with the projected timing of onset of a set frequency (e.g. 2×, 3×, 5× per decade or annual) of disease-conducive conditions. For example, projections have been produced showing the great majority of the world's coral reefs are at risk for coral disease outbreaks before 2050 based on current anthropogenic stress and sea temperature projections [1]. As with the seasonal outlooks, projections can raise awareness among the scientific and management community [8,9], inform planning or build political and social will for future management actions [9,10].

All three types of surveillance tools will produce disease risk forecasts that, as with all forecasts and predictions, inherently have uncertainty. Uncertainty stemming from the strength of the temperature–disease association can be formally acknowledged when sharing tools or tools can be shared with a limited group until research advances. Sometimes, there will be spatial or temporal variation in uncertainty in forecasts that can be qualified or quantified, for example owing to spatial variability in the amounts, types or quality of the information used to generate the forecast. In these cases, Bayesian modelling can be used to produce disease risk forecasts, and a likelihood curve can be presented alongside the prediction, increasing the transparency of resultant research, monitoring or management decisions. Further, local adaptation of the host or parasite, variation in community compositions and host and parasite behaviour and density are all potential drivers of spatial variation in temperature–disease relationships and will change through time. Spatial variation in temperature–disease relationships can be built into surveillance tools using site or region-specific algorithms to forecast disease-promoting conditions, as is possible for coral bleaching [57]. Forecasts of disease risk should not be produced outside the range of the fitted relationship between temperature and disease or for areas where temperature–disease relationships are unknown or poorly understood. Because the temperature–disease relationship will change through time, the statistical models that underpin surveillance tools have to be re-visited regularly as new observations become available, followed by further model validation.

## 3. Case study: epizootic shell disease in the American lobster

**Figure 3.**
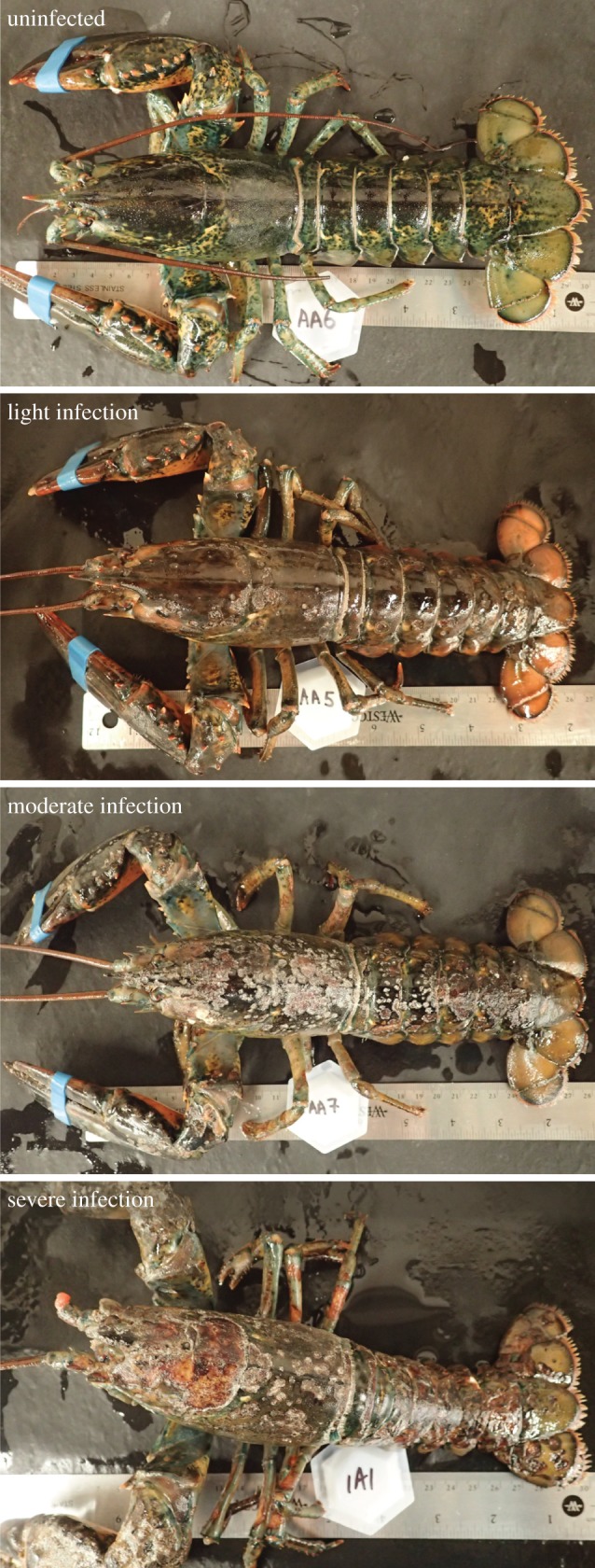
Examples of the American lobster, *H. americanus*, affected by epizootic shell disease (ESD). ESD is characterized by extensive necrosis of the cuticle and surrounding cuticular tissues as chitinoblastic and other bacteria colonize the shell. Severity of the infection varies greatly depending on maturity of the animals, which drives intermoult duration, and the local temperature conditions and water quality. Severely infected animals die owing to the disease. Even animals with light infections on the carapace are not marketable in the lucrative live trade so have less than 10% the value of a healthy animal.

We have undertaken the development process for ESD in the American lobster (figure 3) and have produced initial (‘beta’) versions of all three types of temperature-based surveillance tools. ESD was selected from among the ‘good’ candidate host–pathogen systems because the American lobster is an iconic species with high ecological as well as economic and social/cultural value. Further, fisheries managers confirmed surveillance tools would be of value to a management community gravely concerned about current ESD prevalence levels and potential increases in prevalence or expansion into new areas under climate change.

### (a) Background and aetiology

**Figure 4.**
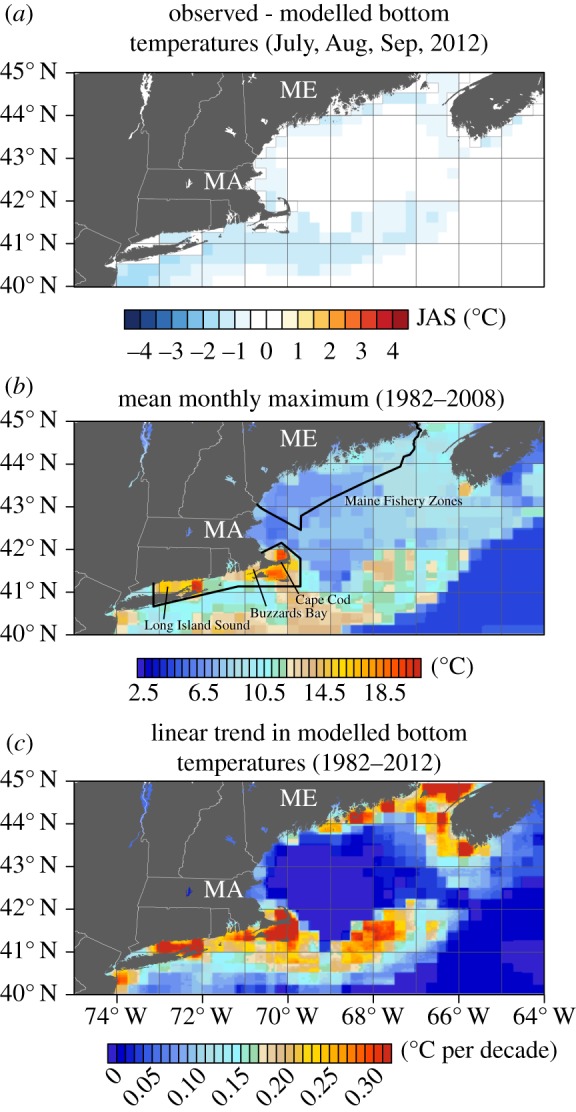
Maps describing aspects of the product development process for the initial versions of the lobster shell disease surveillance tools presented in figure 5. Performance of our modelled bottom temperatures is shown in (*a*); our modelled bottom temperatures are cooler and within 1.5°C (usually less) of observed bottom temperatures from the World Oceans Analysis dataset. The maximum of the monthly mean (MMM) values are shown in (*b*); the lowest MMM in the area where ESD prevalence is more than 5% is 12°C and MMM values in Maine were 7–11°C for the study period. The linear trend in modelled bottom temperatures is shown in (*c*); rates of temperature increase range from 0 to more than 0.3°C per decade.

The American lobster is an iconic species in the coastal New England states (USA) and the Canadian Maritime Provinces. It supports large fisheries with annual landings approaching US$ 1 billion split between Canada and the USA [61,62]. The lobster population off southern New England (SNE) is under severe stress from a combination of increasing ocean temperatures and commercial exploitation [63–65]. The temperature stress in SNE has been linked to an unusual syndrome known as ESD, characterized by the rapid degradation of the ‘shell’ or cuticle [37,66,67]. The disease appeared in eastern Long Island Sound (LIS), Block Island Sound and Buzzards Bay in the late 1990s (see figure 4*b* for locations), and quickly rose in prevalence to around 25–35%, with prevalence levels double that in ovigerous females [68]. There is concern that the disease may be spreading into the highly productive fishery in the Gulf of Maine. Currently, prevalence levels are less than 2% (maximum seen) throughout the shallow coastal waters around the Gulf of Maine and are typically much less than 0.5%.

ESD is an environmental disease involving increased temperature, widespread levels of contaminants and a dysbiosis of the bacterial flora on the cuticle. Chitinoclastic bacteria, notably *Aquimarina homari*, as well as other bacterial species, colonize the cuticle and burrow into it causing extensive necrosis to the surrounding cuticular tissues [45,46]. Our current understanding of ESD aetiology is that increased temperature and contaminants negatively affect host defensive responses, weakening the cuticle and making it more susceptible to the dysbiotic bacterial community [69–73]. ESD is correlated with temperature, and begins to emerge in lobsters when mean annual bottom temperatures rise above 8–10°C or with maximum monthly mean (MMM) bottom temperatures of greater than or equal to 12°C. The disease is prevalent when MMM bottom temperatures approach 20°C [37]. One laboratory study has examined the effect of temperature and bacterial challenges in lobsters [74]. The characteristic lesions of ESD developed at 10°C, but took longer to develop at that temperature and showed less severe histopathology than lesions in animals held at 15 and 20°C [74]. Lobsters are able to shed the infected carapace during moulting, but the new carapace can rapidly become re-infected [75,76]. Importantly, this complicated aetiology has not been fully elucidated, but temperature is a strong component [38].

The direct effect of ESD is that infected lobsters are not marketable in the lucrative live trade owing to extensive necrosis of the carapace and claws (figure 3). Instead, their meat is processed in the canned meat trade, which does not provide sufficient income to cover a fisher's operating costs. More importantly, mortality from ESD is linked to intermoult duration [77], and ESD is more prevalent in ovigerous females that moult less frequently [38]. Once females reach sexual maturity their growth rate slows (measured as an increase in intermoult duration) [78]. Moreover, ovigerous females moult less frequently while they bear eggs, and thus suffer increased mortality from the disease because they cannot moult out of it and often die [38]. The resulting loss in egg production limits juvenile recruitment [79].

Declines in the New England lobster stock have been caused by a suite of stressors (temperature, overfishing, ESD) and have been so drastic that management agencies proposed a moratorium to the fishery off SNE in 2010 [64]. The moratorium was not imposed, and the fishery has since collapsed in the near shore areas. From the disease perspective, there are now two concerns, posed here as questions. First, will the disease continue unabated in SNE, effectively conspiring with other stressors to keep the stock at low population levels? Second, will waters warming under climate change in the northern Gulf of Maine and Nova Scotia cause the disease to increase in prevalence there, affecting the viability of those lobster fishing industries? We use long-term climate model projections to shed light on the answers to these questions. We also develop beta versions of near real-time monitoring and seasonal outlook tools enabling researchers and managers to assess and monitor ESD-conducive conditions. There is a lag time between temperatures and increased prevalence of ESD, so the near real-time monitoring and seasonal outlooks can serve as an early warning system. If the surveillance tools indicate ESD-conducive conditions are present, scientists and managers can increase efforts to monitor ESD. These increased and targeted monitoring efforts can increase understanding of the temperature–disease relationship, which is our goal in developing these initial versions of temperature-based surveillance tools for ESD. For instance, in fisheries models, background mortality and the value of the landed catch are critical inputs for determining catch limits and closures. Being able to predict these under conditions that increase ESD could, therefore, lead to more effective lobster management.

### (b) Developing surveillance tools for epizootic shell disease-conducive sea temperatures

#### (i) Product development methods

We generated a model that compares surface temperatures to the temperatures on the ocean bottom where lobsters reside in a study area inclusive of LIS (southwest corner) and southern Nova Scotia (northeast corner; map extent is 40–45° N, 64–75° W). We used 4-km remotely sensed SST data from the NOAA Pathfinder v5.2 dataset (the US NOAA official climate data record for SST [80]) in combination with the World Oceans Atlas (WOA) [81], which has temperatures at various depths (0.25° resolution). To model bottom temperatures, we calculated a linear regression between monthly mean climatology surface and bottom temperatures in the WOA. We then used these relationships to model bottom temperatures from the observed Pathfinder surface temperatures. Modelled bottom temperatures were not ground-truthed to actual bottom temperatures from sensors for these initial versions of the surveillance tools (i.e. only the Pathfinder to WOA data comparison was undertaken). Modelled bottom temperatures for July–September 2012 were slightly cooler (but within 1.5°C) than observed temperatures in the near shore areas where most of the lobsters reside and were within 0.5°C for the offshore areas (figure 4*a*).

Wahle *et al.* [79] found that the abundance of lobster pre-recruits (sublegal individuals) started to decline when the prevalence of ESD exceeded 5%. The area of SNE in which ESD prevalence has been more than 5% during at least the last 5 years has a polygon around it in figure 4*b*. The MMM climatology values for modelled bottom temperature were calculated and the lowest MMM value that was associated with prevalence values greater than 5% was 12°C. This suggests that the absolute temperature threshold for increased ESD prevalence is a minimum of 12°C, but it is likely to be higher as our modelled bottom temperatures are slightly cool. Glenn & Pugh [37] found ESD to be clearly correlated with temperature and associated with long-term average maximum temperatures (climatology) greater than or equal to 12°C, and there is experimental evidence that lesions develop far more quickly at 15°C than at 10°C [74]. The MMM values for the Gulf of Maine in the fishery reporting grid area are 7–11°C. The required length of exposure to temperatures greater than or equal to 12°C for ESD prevalence to increase is unknown, and is the subject of on-going laboratory experiments; i.e. the shape of the relationship between temperature and ESD development is unknown. Our use of the 12°C threshold represents application of the precautionary principle because it errs on the side of the tools producing false-positives rather than producing false-negatives. This reduces cost-effectiveness of the targeted monitoring somewhat (monitoring places the disease does not occur), but helps ensure the disease is not missed. For the initial versions of temperature-based surveillance tools, we assumed that modelled bottom temperatures need only exceed 12°C for a day for the near real-time monitoring tool, a week for the seasonal outlook, and a month for the long-term projections. These timeframes are based on the temporal resolution of the temperature data used to develop each product type.

Our real-time monitoring of SST in the area of interest is based on the 5-km CRW product described in Liu *et al.* [7] and is presented for 15 September 2014. The seasonal outlook produced has a 3-month lead-time and presents the probabilistic outlook for September 2015 of bottom temperatures exceeding 12°C for a minimum of seven consecutive days based on model runs from 22–28 June 2015. To generate the seasonal outlook, we calculated the SST values required for modelled bottom temperatures of 12°C (see electronic supplementary material, figure S1), and then used the SST forecast from the NOAA National Center for Environmental Prediction's (NCEP) Climate Forecast System Version 2 (CFSv2) (see electronic supplementary material for additional methods for the seasonal outlook). The long-term outlooks are statistically downscaled (4-km resolution) climate model ensemble-based projections for the fossil-fuel aggressive emissions scenario RCP8.5, following van Hooidonk *et al.* [10] (see electronic supplementary material, table S2 for models list). The projections produced are for the timing of the onset of maximum temperatures greater than or equal to 12°C (i.e. annual exceedance).

#### (ii) Product development results

**Figure 5.**
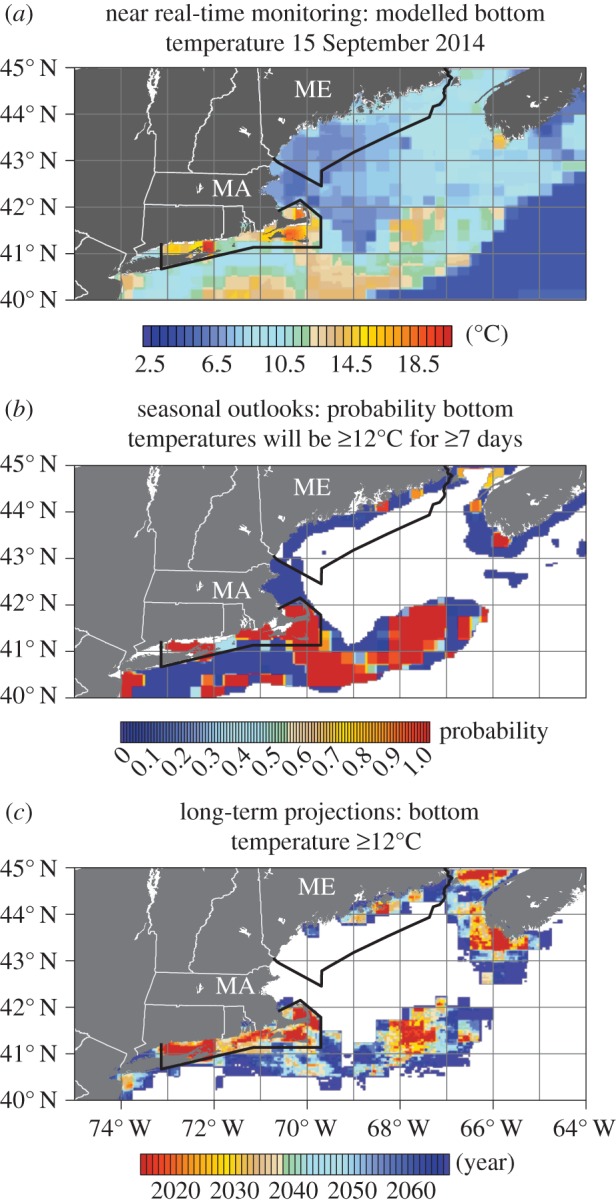
Initial versions of three surveillance tools developed for lobster shell disease. Near real-time monitoring (*a*) for 15 September 2014 shows modelled bottom temperatures (based on satellite SST) were greater than or equal to 12°C in southern New England and less than 12°C in the northern Gulf of Maine. The seasonal outlook (*b*) for September 2015 as of June 2015 suggested temperatures would be greater than 12°C in parts of the northern Gulf of Maine in 2015 (90+% probability). The long-term projections (*c*) suggest bottom temperatures will be greater than or equal to 12°C in the next 20 years in more than half of the coastal fishery in the northern Gulf of Maine and for southern coastal Nova Scotia. Data are only shown for (*b*) and (*c*) for depths less than 100 m.

The near real-time monitoring for 2014 suggests modelled bottom temperatures are regularly more than 12°C in SNE and regularly approach 20°C where prevalence of ESD currently ranges from 10 to 40% (figure 5) [67,68]. Modelled bottom temperatures in the Gulf of Maine fishery reporting grid area were 7–11°C at this time. September is the month in which modelled bottom temperatures have historically been highest for most of the area of interest; hence, temperatures are unlikely to have been much higher in the preceding or following month.

For SNE, the seasonal outlook for September 2015, as of June 2015, suggests bottom temperatures in 2015 will equal or exceed what was observed mid-September in 2014. For the northern Gulf of Maine, if the bottom temperatures forecasted in the seasonal outlook manifest, 2015 will be the warmest year in the last 30 years in some of the areas where Maine lobsters are fished. These temperatures could lead to increased ESD prevalence in the northern Gulf of Maine in 2016. This result demonstrates the value of seasonal outlooks for targeting monitoring efforts. For example, bottom temperatures will be monitored through 2016 to ground-truth the surface–bottom relationship in the seasonal outlook algorithm and to compare ESD prevalence in 2016 with other recent years.

The areas within the Gulf of Maine where bottom temperatures are forecasted to be more than 12°C for at least seven consecutive days in 2015 are the areas where the rates of increase in bottom temperatures from 1982 to 2008 were greatest (figure 4*c*). Linear trends in bottom temperature range in the area of interest from zero to more than 0.3°C per decade. The highest rates of increase are throughout the SNE area where ESD prevalence is and has been greater than 5% (last approx. 10 years) and in parts of the Gulf of Maine and western and southern Nova Scotia. The long-term projections suggest past trends of temperature increase in these areas will continue. Maximum temperatures are projected to be more than 12°C annually within the next 20 years (figure 5*c*) in most of the Gulf of Maine fishery zones (especially east) and in western and southern Nova Scotia. Currently, prevalence levels of ESD are higher in the western (approx. 2%) than in the eastern Gulf of Maine (less than 0.5%, K.R., unpublished data). The long-term projections suggest this may change as temperatures have been (figure 4*c*) and are projected to warm more rapidly in the eastern than western portions of the Gulf (figure 5*b*). These projections are conservative; the projected rates of temperature increase for the coming decades in the area of interest are less than has been observed across the three recent decades (electronic supplementary material, figure S2).

#### (iii) Future research

Results from experiments as well as additional field observations from on-going monitoring programmes in Maine can be used to further refine the versions of the surveillance tools presented here. The prevalence of ESD (usually less than 0.5%) has been too low in the fishing area off Maine (i.e. low prevalence and temperatures less than 12°C) to use existing data to determine whether temperature thresholds for ESD are different there than for lobsters off SNE. Consequently, the general belief is that bottom temperatures have thus far been too low in the Gulf of Maine to facilitate the emergence of ESD. As yet, very few laboratory experiments have been conducted on the progression of ESD in relation to temperature. The few experiments that have been conducted have used lobsters caught in SNE or reared in aquaria. The population of lobsters in the Gulf of Maine is likely to be locally acclimated and genetically adapted; thus, the suite of conditions facilitating ESD (temperature included) will probably be different there than in SNE. Future experiments could examine whether rates of progression of ESD differ between lobsters from the northern Gulf of Maine and lobsters from SNE. Results from experiments can then be combined with monitoring efforts at ground-truth predictions from the surveillance tools.

## 4. Conclusion

As feared, recent increases in temperatures thought to have contributed to ESD onset and rapid progression in SNE are projected to continue unabated. Sea surface (and modelled bottom) temperatures are projected to increase at faster rates in SNE than anywhere else in the Gulf of Maine or Nova Scotia. Our projections indicate that high prevalence levels of ESD are likely to persist in SNE. Concerns related to ESD prevalence increase and ESD expansion in the northern Gulf of Maine also seem warranted but require further research and clarification. Recent bottom temperature increases coincide with recent increases in ESD prevalence in parts of the Maine fishery; however, prevalence is still less than 2% (K.R., unpublished data). Along with the results presented here, recent increases in ESD prevalence in Maine may continue in coming years, especially in shallow bays where waters are warming most quickly. The experiments we describe above can help determine whether substantial increases in ESD prevalence are likely to accompany the projected increases in temperature maxima.

Iterative refinement is a key feature of the development process for surveillance tools. Product deployment, including the sharing of initial versions of tools (as is the case here), is a process rather than an endpoint. There are no host–disease–temperature relationships in the marine environment for which our understanding of the aetiology is exhaustive and host–disease–temperature relationships may change over time. Further, there are various other environmental parameters (e.g. salinity, water quality) that may influence the incidence of marine disease; where appropriate, these should be incorporated into more sophisticated tools that consider multiple environmental stressors simultaneously.

Temperature-based disease surveillance tools are needed, because outbreaks of diseases known to increase with warming are likely to increase in frequency and severity as waters warm under climate change. Meeting the increasing need for these tools is possible if the appropriate research data, resources and assessment tools can be brought together. New legislative frameworks could provide resources for developing forecasting tools to the extent required to manage marine disease outbreaks [80]. For example, successful passing of the Marine Disease Emergency (MDE) Act in the USA, under consideration as of December 2015, would ensure marine disease outbreaks are considered for classification as ‘emergencies' [82,83]. The MDE Act would also establish central data repositories that will aid in developing more surveillance tools by increasing the accessibility of data on disease observations. That an MDE Act is being considered is indicative of the inertia behind the idea that marine disease outbreaks warrant well-resourced responses. The temperature-based disease surveillance tools we describe here can inform these strategic responses, increasing our ability to adaptively manage disease and downstream impacts.

## Supplementary Material

Supplementary material

## Data accessibility

The data used to produce figures 4 and 5 are available by sending a request to the corresponding author.
