# Supplementary material for: Improving marine disease surveillance through sea temperature monitoring, outlooks and projections
Source: Philos Trans R Soc Lond B Biol Sci. 2016 Mar 5;371(1689):20150208. doi: 10.1098/rstb.2015.0208 (PMC4760138; doi:10.1098/rstb.2015.0208)
Supplement: Supplementary material [file rstb20150208supp1.pdf]

Phil. Trans. R. Soc. B doi: 10.1098/rstb.2015.0208

Supplementary material

Improving marine disease surveillance through sea temperature monitoring, outlooks and projections

Jeffrey Maynard*, Ruben van Hooidonk, C. Drew Harvell, C. Mark Eakin, Gang Liu, Bette L. Willis, Gareth J. Williams, Maya Groner, Andrew Dobson, Scott F. Heron, Robert Glenn, Kathleen Reardon, Jeffrey D. Shields

*Corresponding author: E: maynardmarine@gmail.com

Contents

1. Detailed methods for seasonal outlook presented as Figure 5b.
2. Table S1 – Marine host-pathogen systems that are potential candidates for temperature-based disease surveillance tools.
3. Table S2. List of CMIP5 climate models forced with RCP8.5 to produce the long-term projections presented as Figure 5c.
4. Figure S1. Required surface temperatures for modeled bottom temperatures of 12 °C.
5. Figure S2. Comparison of linear trends in SST; remote sensing vs. climate model projections.
6. References

1. Detailed methods for seasonal outlook presented as Figure 5b.

The SST forecast used in the beta version of the seasonal outlook presented as Figure 5b is the 9-month daily temperature forecast for the sea surface layer (top 10 meters) from the NOAA National Center for Environmental Prediction's (NCEP) Climate Forecast System Version 2 (CFSv2) model (Saha et al. 2014; CFSv2 website: http://cfs.ncep.noaa.gov). CFSv2 became operational in March 2011. CFSv2 runs four times every day at 0000, 0600, 1200, and 1800 UTC producing forecast for each future day up to 9 months into the future.

CFSv2 is a fully coupled ocean–land–atmosphere dynamical seasonal prediction system. For the study area, CFSv2 has both zonal and meridional resolutions of 0.5°. The ocean has 40 vertical layers in the model with 27 layers in the upper 400 m, and the bottom depth is around 4.5 km. The vertical resolution is 10 m from the surface to 240-m depth.

For this study, nine-month daily SST forecasts from each of the CFSv2 nine-month model runs that occurred from June 22 through 28 (Monday-Sunday) 2015 were examined. For each data grid, we determined whether the predicted sea surface temperatures reach or exceed the surface temperatures required for modeled bottom temperatures to be ≥12 °C at the grid (see Figure S1) for at least seven consecutive days in September, 2015. Collecting the duration predictions for September 2015 from all four model runs per day over the 7-day period of June 22-28 produced a set of 28 runs (i.e., 28 ensemble members). We then generated a probabilistic forecast for each grid by calculating the percentage of the 28 runs that predicted the occurrence of bottom temperature ≥12 °C for all seven days.

1. Table S1. Host-pathogen systems identified as potential candidates for developing temperature-based surveillance tools. For these systems, temperature is strongly suspected as having a role in the disease etiology but the strength and nature of the role is not well understood. This list shows the breadth of host-pathogen systems that may eventually be good candidates for surveillance tools but is not expected to be exhaustive.

Hosts	Species	Causative agent or disease name	Region	References
**Sponges**				
Vase sponge	Ircinia spp.	*Vibrio spp.*	Mediterranean Sea	Maldonado et al. (2010); Stabili et al. (2012)
Sponges	*Spongia spp., Hippospongia spp.*	*Microbial consortium*	Mediterranean Sea	Gaino et al. (1992)
**Corals**				
Sea fans	Gorgonia	*Aspergillus sydowii*	Caribbean Sea	Alker et al. (2001)
Sea fans	Gorgonia	*mortality*	Caribbean Sea	Cerrano et al. (2000)
**Molluscs**				
Pacific oyster	*Crassostrea gigas*	*Oyster herpes virus*	W Europe, W USA	Le Deuff et al. (1996); Burge et al. (2007)
European oyster	*Ostrea edulis*	*Bonamia ostreae*	Western Europe	Van Banning (1991); Engelsma et al. (2010)
European oyster	*Ostrea edulis*	*Marteilia refringens*	Western Europe	Balouet et al. (1979); Alderman (1979); Audemard et al. (2004)
Abalone	*Haliotis spp.*	*Xenohaliotis haliotidis*	Western USA	Friedman et al. (1997); Braid et al. (2005)
**Crustaceans**				
Shrimp	Penaeid shrimp	*White Spot Syndrome*	Global	Rahman et al. (2006); Rahman et al. (2007)
Shrimp	Penaeid shrimp	*IHHNV*	Global	Montgomery-Brock et al. (2007)
Blue crab	*Callinectes sapidus*	*Hematodinium perezi*	Mid-Atlantic USA	Messick et al. (1999), Messick & Shields (2000)
Spiny lobster	*Panulirus argus*	*PaV1*	Caribbean Sea	Behringer et al. (2008); Behringer et al. (2012)
**Echinoderms**				
Sea urchin	*Str. droebachensis*	*Paramoeba*	Northeast Canada	Scheibling & Hennigar (1997); Buchwald et al. (2015)
Sea stars	Sea stars - 22 species	*Sea Star Wasting Disease*	E and W USA and Canada	Eisenlord et al. (this issue); Hewson et al. (2014)
**Vertebrates**				
Salmon	Salmonids	*IHNV*	Pacific and Atlantic-coastal Japan, W and E US, W Canada, Europe	Garver et al. (2013)

4. Table S2. List of CMIP5 climate models forced with RCP8.5 to produce the long-term projections presented as Figure 5c.

Model name	Model name	Model name
CCSM4	MIROC5	CMCC-CMS
IPSL-CM5A-LR	GFDL-CM3	EC-EARTH
BCC-CSM1.1	CNRM-CM5	FIO-ESM
MRI-CGCM3	NorESM1-M	HadGEM2-AO
IPSL-CM5A-MR	ACCESS1-0	INM-CM4
CSIRO-Mk3.6.0	ACCESS1-3	IPSL-CM5B-LR
GFDL-ESM2G	BCC-CSM1.1(m)	MPI-ESM-LR
GISS-E2-R	CESM1-BGC	MPI-ESM-MR
HadGEM2-CC	CESM1-CAM5	NorESM1-ME
GFDL-ESM2M	CESM1-ACCM	
HadGEM2-ES	CMCC-CESM	
CanESM2	CMCC-CM	

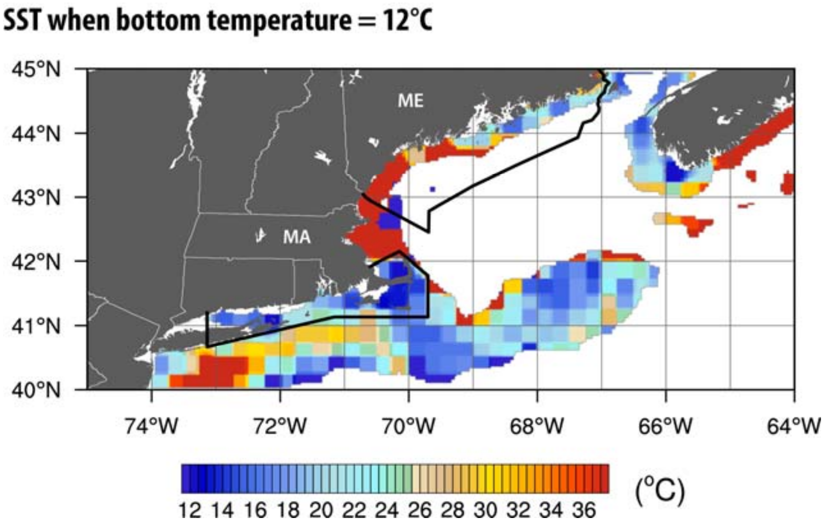

3. Figure S1. Required surface temperatures for modeled bottom temperatures of 12 °C. Relationships between surface and bottom temperatures were derived from a linear regression relating remotely sensed SST (NOAA Pathfinder v5.2) to World Oceans Analysis data for July-August, 2012 (see methods in paper).

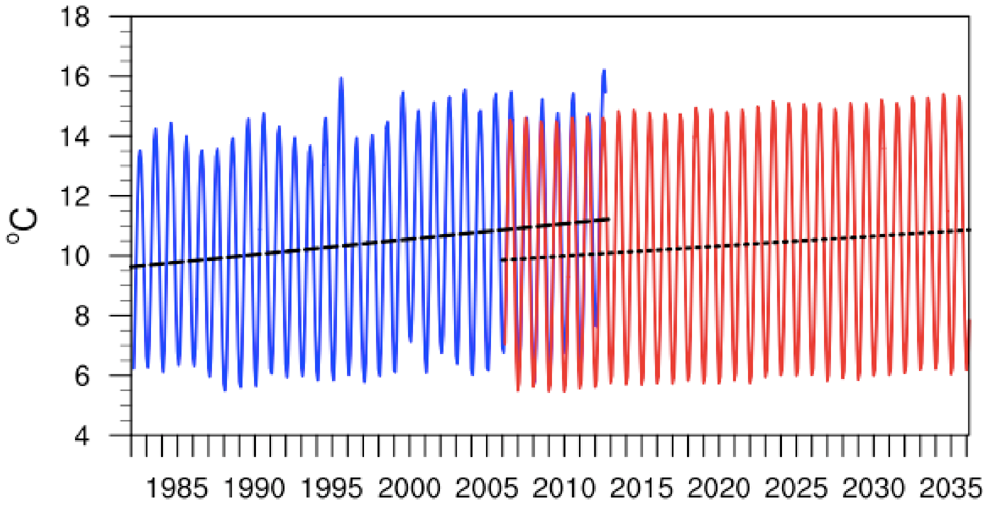

5. Figure S2. Linear trend in sea surface temperatures (SST) averaged across the spatial domain shown in Figures 4 and 5 for remotely sensed SST (blue, NOAA Pathfinder v5.2) and the CMIP5 climate model ensemble used for the long-term projections (red). We state in the paper that the long-term projections are likely conservative because they are based on rates of change in SST for the next 20 years that are less than what was documented (on average) these last 30 years.

6. References

Alderman DJ. 1979 Epizootiology of Marteilia refringens in Europe. *Mar. Fish. Rev.* **41**(1-2), 67-69.

Alker AP, Smith GW, Kim K. 2001 Characterization of Aspergillus sydowii (Thom et Church), a fungal pathogen of Caribbean sea fan corals. *Hydrobiologia*. **460**(1-3), 105-111.

Audemard C, Sajus MC, Barnaud A, Sautour B, Sauriau PG, Berthe F. 2004 Infection dynamics of Marteilia refringens in flat oyster Ostrea edulis and copepod Paracartia grani in a claire pond of Marennes-Oleron Bay. *Dis. Aquat. Organ.* **61**(1-2), 103-111.

Balouet G. 1979 Marteilia refringens—considerations of the life cycle and development of Abers disease in Ostrea edulis. *Mar. Fish. Rev.* **41**(1-2), 64-66.

Behringer DC, Butler IV MJ, Moss J, Shields JD. 2012 PaV1 infection in the Florida spiny lobster (Panulirus argus) fishery and its effects on trap function and disease transmission. *Can. J. Fish Aquat. Sci.* **69**(1), 136-144.

Behringer DC, Butler MJ, Shields JD. 2008 Ecological and physiological effects of PaV1 infection on the Caribbean spiny lobster (Panulirus argus Latreille). *J. Exp. Mar. Bio. Ecol.* **359**(1), 26-33.

Braid BA, Moore JD, Robbins TT, Hedrick RP, Tjeerdema RS, Friedman CS. 2005 Health and survival of red abalone, Haliotis rufescens, under varying temperature, food supply, and exposure to the agent of withering syndrome. *J. Invertebr. Pathol.* **89**(3), 219-231.

Buchwald RT, Feehan CJ, Scheibling RE, Simpson AG. 2015 Low temperature tolerance of a sea urchin pathogen: Implications for benthic community dynamics in a warming ocean. *J. Exp. Mar. Bio. Ecol.* **469,** 1-9.

Burge CA *et al.* 2014 Climate change influences on marine infectious diseases: implications for management and society. *Ann. Rev. Mar. Sci.* **6,** 249-277.

Cerrano C *et al.* 2000 A catastrophic mass-mortality episode of gorgonians and other organisms in the Ligurian Sea (North-western Mediterranean), summer 1999. *Ecol. Lett.* **3**(4), 284-293.

Eisenlord M *et al.* in review this issue Title unknown at time of submission; will complete reference at R1.

Engelsma MY *et al.* 2010 Epidemiology of Bonamia ostreae infecting European flat oysters (Ostrea edulis) from Lake Grevelingen, The Netherlands. *Mar. Ecol. Prog. Ser.* **409**, 131-142.

Friedman CS, Thomson M, Chun C, Haaker PL, Hedrick RP. 1997 Withering syndrome of the black abalone, Haliotis cracherodii (Leach): water temperature, food availability, and parasites as possible causes. *J. Shellfish Res.* **16**(2), 403-412.

Gaino E, Pronzato R, Corrier G, Buffa P. 1992 Mortality of commercial sponges: incidence in two Mediterranean areas. *Ital. J. Zool.* **59**(1), 79-85.

Garver KA, Mahony AA, Stucchi D, Richard J, Van Woensel C, Foreman M. 2013 Estimation of parameters influencing waterborne transmission of infectious Hematopoietic Necrosis Virus (IHNV) in Atlantic Salmon (Salmo salar). *PLoS One* (doi: 10.1371/journal.pone.0082296)

Hewson I *et al.* 2014. Densovirus associated with sea-star wasting disease and mass mortality. *Proc. Natl. Acad. Sci. USA* **111(4**8), 17278-17283.

Le Deuff RM, Renault T, Gerard A. 1996 Effects of temperature on herpes-like virus detection among hatchery-reared larval Pacific oyster Crassostrea gigas. *Dis. Aquat. Organ.* **24**(2), 149-157.

Maldonado M, Sánchez-Tocino L, Navarro C. 2010 Recurrent disease outbreaks in corneous demosponges of the genus Ircinia: epidemic incidence and defense mechanisms. *Mar. Biol.* **157**(7), 1577-1590.

Messick GA, Shields JD. 2000 Epizootiology of the parasitic dinoflagellate Hematodinium sp. in the American blue crab Callinectes sapidus. *Dis. Aquat. Organ.* **43**(2), 139-152.

Messick GA, Jordan SJ, Heukelem WFV. 1999 Salinity and temperature effects on Hematodinium sp in the blue crab *Callinectes sapidus*. *J. Shellfish Res.* **18**(2), 657-662.

Montgomery-Brock D, Tacon AG, Poulos B, Lightner D. 2007 Reduced replication of infectious hypodermal and hematopoietic necrosis virus (IHHNV) in Litopenaeus vannamei held in warm water. *Aquaculture* **265**(1), 41-48.

Rahman M *et al.* 2006 Effect of high water temperature (33 C) on the clinical and virological outcome of experimental infections with white spot syndrome virus (WSSV) in specific pathogen-free (SPF) Litopenaeus vannamei. *Aquaculture* **261(**3), 842-849.

Rahman MM, Corteel M, Wille M, Alday-Sanz V, Pensaert MB, Sorgeloos P, Nauwynck HJ. 2007 The effect of raising water temperature to 33 C in Penaeus vannamei juveniles at different stages of infection with white spot syndrome virus (WSSV). *Aquaculture* **272**(1), 240-245.

Saha S *et al.* 2014 The NCEP climate forecast system version 2. *J. Clim.* **27**(6), 2185-2208.

Scheibling RE, Hennigar AW. 1997 Recurrent outbreaks of disease in sea urchins Strongylocentrotus droebachiensis in Nova Scotia: evidence for a link with large-scale meteorologic and oceanographic events. *Mar. Ecol. Prog. Ser.* **152**(1), 155-165.

Stabili L, Cardone F, Alifano P, Tredici SM, Piraino S, Corriero G, Gaino E. 2012 Epidemic mortality of the sponge Ircinia variabilis (Schmidt, 1862) associated to proliferation of a Vibrio bacterium. *Microb. Ecol.* **64**(3), 802-813.

Van Banning P. 1991 Observations on bonamiasis in the stock of the European flat oyster, Ostrea edulis, in the Netherlands, with special reference to the recent developments in Lake Grevelingen. *Aquaculture*, *93*(3), 205-211.
